# Quality Characteristics of Flavored Butter Supplemented with Yuzu Powder and Almond Granules

**DOI:** 10.3390/foods15193531

**Published:** 2026-10-03

**Authors:** Ga-eun Kim, Hyeon-seo Lee, Yun-seo Bong, Yeon-Su Jeong, Sin-Young Park

**Affiliations:** 1 Department of Animal Resource Science, Kongju National University, Chungnam 32439, Republic of Korea; 2 Resources Science Research Institute, Kongju National University, Chungnam 32439, Republic of Korea

**Keywords:** flavored butter, almond, yuzu, physicochemical properties, sensory properties

## Abstract

This study evaluated the quality characteristics of flavored butter containing a fixed level of yuzu powder and varying levels of almond granules. Samples were prepared with 1% yuzu powder and 3%, 6%, or 9% almond granules (CA3, CA6, and CA9, respectively), alongside an unsupplemented control (Con). Moisture, fat, and ash contents, active acidity (pH), color, viscosity, volatile profile patterns, instrumental taste profiles, and sensory attributes were evaluated. All supplemented samples had lower moisture contents than Con, whereas CA3 showed the highest fat content. Con showed the highest ash content; among the supplemented samples, CA3 had a higher ash content than CA6 and CA9, which did not differ significantly. Viscosity decreased as the almond level increased under the fixed yuzu concentration. Electronic-tongue analysis showed distinct taste profiles among the formulations, with the sourness response decreasing in the order Con > CA9 > CA6 > CA3. Electronic-nose analysis showed that the supplemented formulations had volatile profile patterns distinct from Con, although individual compounds were not definitively identified. CA3 showed favorable flavor and taste scores, whereas CA3 and CA6 exhibited comparable overall acceptability. These findings suggest that lower-to-moderate almond levels may provide a favorable sensory balance in flavored butter containing 1% yuzu powder. However, the independent effects of yuzu powder and almond granules could not be determined because single-ingredient control groups were not included.

## 1. Introduction

Dairy products have long been consumed as major dietary sources of protein and fat, and butter and cream in particular occupy an important position because of their flavor and versatility in culinary applications. Butter is a representative dairy product manufactured by separating or fermenting milk fat from raw milk obtained from dairy animals, such as cows and dairy goats, followed by churning and working [1]. However, the Korean dairy processing industry relies heavily on imported dairy ingredients for the manufacture of products such as cheese and butter [2]. Rising domestic raw milk prices and the consequent increase in the prices of domestically produced dairy products have increased the consumption of products manufactured from imported ingredients, which now account for more than half of the domestic market [3]. Moreover, butter products manufactured and marketed by Korean companies consist predominantly of conventional natural and processed butter. Other categories, including cultured and flavored butter, depend largely on imports; consequently, the domestic dairy market offers substantially less product diversity than overseas markets [4].

Nevertheless, dairy consumption in Korea has increased steadily over the past decade, and butter consumption has continued to rise in recent years [5]. As this growth has coincided with a stronger consumer emphasis on product quality, manufacturers have sought to develop more diverse, high-quality butter products [6]. Consumers have also shown interest in dairy products formulated with diverse ingredients and sensory characteristics [7].

Accordingly, flavored butter development may benefit from ingredients that provide distinctive aroma, taste, texture, and appearance. Almonds contain protein, lipids, vitamin E, minerals, and dietary fiber and are rich in monounsaturated and polyunsaturated fatty acids [8,9]. They also impart nutty sensory characteristics when incorporated into foods [10]. The previously reported nutritional properties of almonds provide background for their use as a food ingredient but were not evaluated in the present study. Reflecting their commercial relevance, the global nut butter market increased from USD 3.55 billion in 2023 to USD 3.70 billion in 2024 and is projected to reach USD 5.04 billion by 2032 [11].

Yuzu (*Citrus junos*) is a citrus fruit cultivated primarily in Korea and Japan, and its peel and pulp are used in diverse food products [12]. Previous studies have reported that yuzu contains vitamin C, organic acids, polyphenols, limonoids, and other antioxidant constituents [13,14,15]. These reported compositional and functional properties provide background for its use as a food ingredient; however, they were not measured in the present study.

Potential complementary antioxidant effects of vitamins C and E have been discussed in previous studies [16,17]. However, vitamin concentrations and antioxidant activity were not measured in the present study; therefore, no functional or synergistic antioxidant effects were inferred from the current results.

Therefore, this study evaluated the proximate composition, physicochemical properties, instrumental aroma and taste profiles, and sensory characteristics of flavored butter containing a fixed level of yuzu powder (1%) and varying levels of almond granules (3%, 6%, and 9%). An unsupplemented butter was used as the control. The study aimed to characterize differences among the tested formulations and identify a sensorially favorable formulation. Because yuzu-only and almond-only treatments were not included, the study was not designed to determine the independent effects of the two ingredients.

## 2. Materials and Methods

### 2.1. Preparation of Flavored Butter Supplemented with Yuzu Powder and Almond Granules

**Figure 1:**
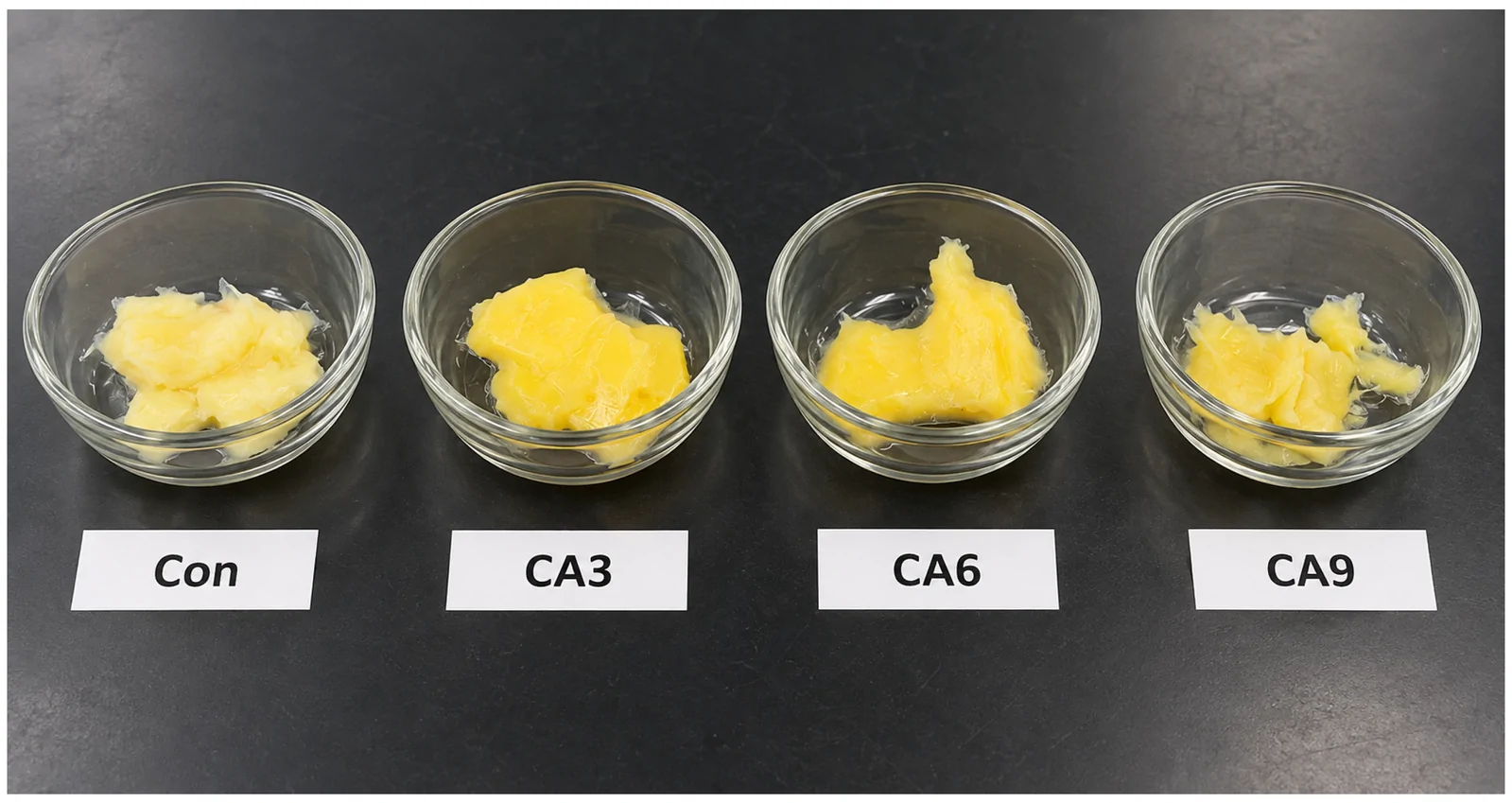
Appearance of flavored butter supplemented with yuzu powder and almond granules. Con: unsupplemented butter; CA3: butter with 1% yuzu powder and 3% almond granules; CA6: butter with 1% yuzu powder and 6% almond granules; CA9: butter with 1% yuzu powder and 9% almond granules.

Commercial whipping cream (Maeil Milk, Seoul, Republic of Korea; milk- fat con-tent: 38%), yuzu powder (Pureunsan Agricultural Corporation Co., Ltd., Seoul, Republic of Korea), and almonds (Jungbu Market, Seoul, Republic of Korea) were used. Al-monds (90 g) were ground for 3 min using a blender (51BL30, Waring Commercial, Torrington, CT, USA). For each treatment, whipping cream (500 mL) was mixed with 1% yuzu powder (5 g/500 mL cream), followed by almond gran-ules at 3% (15 g), 6% (30 g), or 9% (45 g) relative to the initial cream volume. A sample prepared without yuzu powder or almond granules was designated as the unsupple-mented control (Con). Each formulation was churned using a household hand mixer (HR3705/00, Philips, Amsterdam, Netherlands) at 1150 rpm until visible butter grains formed and the aque-ous phase separated. The approximate churning times were 30, 40, 40, and 45 min for Con, CA3, CA6, and CA9, respectively. The butter was maintained in a water bath at 40 °C for approximately 3 min and separated using 30 mesh (Approximately 600 μm) food-grade cheesecloth. The resulting butter was stored at 4 °C for 4 h before analysis. The prepared flavored butter samples are shown in Figure 1.

### 2.2. Proximate Composition Analysis

Moisture, fat, and ash contents were determined according to 15th AOAC methods 932.06 [18]. Moisture was measured by atmospheric-pressure oven drying at 105 °C, fat by Soxhlet extraction, and ash by dry ashing at 550 °C.

### 2.3. Active Acidity (pH) Measurement

Four grams of each sample was mixed with 16 mL of distilled water and homogenized at 2000 rpm for 1 min using an Ultra-Turrax homogenizer (HMZ-20DN, Pooglim Tech, Seongnam, Korea). Active acidity (pH) was measured using a glass-electrode pH meter (S220, Mettler-Toledo, Schwerzenbach, Switzerland). Before measurement, the pH meter was calibrated using standard buffer solutions at pH 4.01, 7.00, and 10.01.

### 2.4. Color Measurement

Color was measured using a colorimeter (CR-10, Minolta, Osaka, Japan) calibrated with a white standard plate (L* = 93.93, a* = -0.43, and b* = 1.98; Y = 87.77, x = 0.347, and y = 0.360). The measured values were expressed as lightness (L*), redness (a*), and yellowness (b*). Chroma (C*ab) and hue angle (h°) were calculated as C*ab = (a*^2^ + b*^2^)^1^ᐟ^2^ and h° = atan2(b*, a*) × 180/π, respectively. Total color difference relative to Con was calculated as ΔE*ab = [(ΔL*)^2^ + (Δa*)^2^ + (Δb*)^2^]^1^ᐟ^2^.

### 2.5. Viscosity Measurement

Each sample was placed in a conical tube to a depth of approximately 10 mm. Viscosity was measured three times per independent batch at 30 rpm for 30 s using a viscometer with spindle No. 4 (WVS-2M, Daihan Scientific, Wonju, Republic of Korea), and the mean value was expressed in mPa·s.

### 2.6. Electronic-Nose Analysis

Volatile profile patterns were analyzed using a Heracles II electronic nose (Alpha MOS, Toulouse, France). Four grams of each sample (Con, CA3, CA6, and CA9) were placed in a 20-mL vial. The analytical conditions were as follows: flow rate, 250 mL/min; analysis time, 110 s; incubation temperature, 60 °C; incubation time, 20 min; and injection volume, 5 mL. Volatile peaks and their relative intensities were displayed as chromatograms using AlphaSoft software ver. 2025-7.3.3 (Alpha MOS). Peaks were tentatively assigned based on retention indices and the instrument library. Because GC-MS, GC-IMS, authentic standards, and quantitative internal standards were not used, the peak assignments were not regarded as definitive compound identification. PCA was conducted using AlphaSoft software to compare volatile profile patterns among samples, which were described using the first and second principal components (PC1 and PC2, respectively). All measurements were performed in triplicate for each independent batch.

### 2.7. Electronic Tongue Analysis

Instrumental taste characteristics were analyzed using an Astree electronic tongue (Alpha MOS, Toulouse, France). Reference solutions of 0.1 M HCl, 0.1 M NaCl, and 0.1 M monosodium glutamate were used for sourness, saltiness, and umami, respectively. Four grams of each sample (Con, CA3, CA6, and CA9) was mixed with 16 mL of distilled water and manually homogenized. The homogenate was filtered through quantitative, medium-hardened filter paper (Filtratech, Paris, France), and the filtrate was diluted 1:100 with distilled water before analysis. Sensor responses were acquired for 120 s using AlphaSoft software (Alpha MOS) and expressed as AHS (sourness), PKS, CTS (saltiness), NMS (umami), CPS, ANS, and SCS. Before each replicate measurement, the sensor array was rinsed with distilled water as a conditioning step to minimize carryover. PCA was performed using AlphaSoft software, and differences among samples were described using PC1 and PC2. All measurements were performed in triplicate for each independent batch.

### 2.8. Sensory Evaluation

The sensory evaluation was conducted as an acceptance test after approval by the Institutional Review Board of Kongju National University (approval code: KNU_IRB 2025-021; formal approval date: 21 May 2025; applicable from 1 January 2025). Eight trained panelists evaluated four flavored butter samples (Con, CA3, CA6, and CA9). Before the formal evaluation, the panelists received training on the definitions of the sensory attributes and the use of the 10-point scale. During the training, the panelists evaluated representative butter samples and discussed each attribute to establish a consistent understanding of the evaluation criteria. Approximately 5 g of each sample was placed in an identical container, labeled with a random three-digit code, and presented in randomized order at 25 °C. Drinking water was provided for palate cleansing between samples. The same panelists completed three independent evaluation sessions. Color, flavor, off-flavor, taste, greasiness, hardness, purchase intent, and overall acceptability were evaluated using a 10-point scale. Color, taste, hardness, and overall acceptability were rated from 1 (extremely unacceptable) to 10 (extremely desirable); flavor was rated from 1 (extremely inadequate) to 10 (extremely adequate); off-flavor was rated from 1 (extremely intense) to 10 (none); greasiness was rated from 1 (extremely weak) to 10 (extremely intense); and purchase intent was rated from 1 (no intention to purchase) to 10 (very willing to purchase).

### 2.9. Statistical Analysis

Each formulation was prepared in three independent batches, and each analysis was performed three times per batch. Data were analyzed using SAS software (version 9.4 for Windows; SAS Institute Inc., Cary, NC, USA) and expressed as the mean ± standard deviation. One-way analysis of variance followed by Duncan’s multiple range test was used to determine differences among treatments. Differences were considered statistically significant at *p* < 0.05, and exact ANOVA *p*-values are reported in the corresponding tables.

## 3. Results and Discussion

### 3.1. Proximate Composition

The proximate compositions of the flavored butter formulations are presented in Table 1.

All supplemented formulations had significantly lower moisture contents than Con (*p* < 0.05). CA3 had a lower moisture content than CA9, whereas CA6 did not differ significantly from CA3 or CA9. The lower moisture contents of the supplemented formulations may reflect compositional differences resulting from the combined addition of yuzu powder and almond granules and differences in aqueous-phase removal during butter manufacture. Although fat content can influence water retention in fat-rich foods [19], this mechanism was not directly examined in the present study. Con had the lowest fat content (*p* < 0.05). Among the supplemented formulations, CA3 and CA6 did not differ significantly, nor did CA6 and CA9; however, CA3 had a higher fat content than CA9. Thus, the supplemented formulations had higher fat contents than Con, but fat content did not increase proportionally with the almond level and was numerically highest in CA3. Almonds contain substantial amounts of lipids [9]; nevertheless, differences between Con and the supplemented formulations should be interpreted as combined formulation and processing effects rather than an independent almond effect. Con had the highest ash content (*p* < 0.05). Among the supplemented formulations, CA3 had a higher ash content than CA6 and CA9, whereas CA6 and CA9 did not differ significantly. Although almonds contain minerals and ash [20], ash content did not increase proportionally with almond level. The observed differences may reflect variation in the relative proportions and retention of milk-derived solids, moisture, fat, and mineral-containing components during churning and buttermilk removal.

### 3.2. Active Acidity (pH)

**Figure 2:**
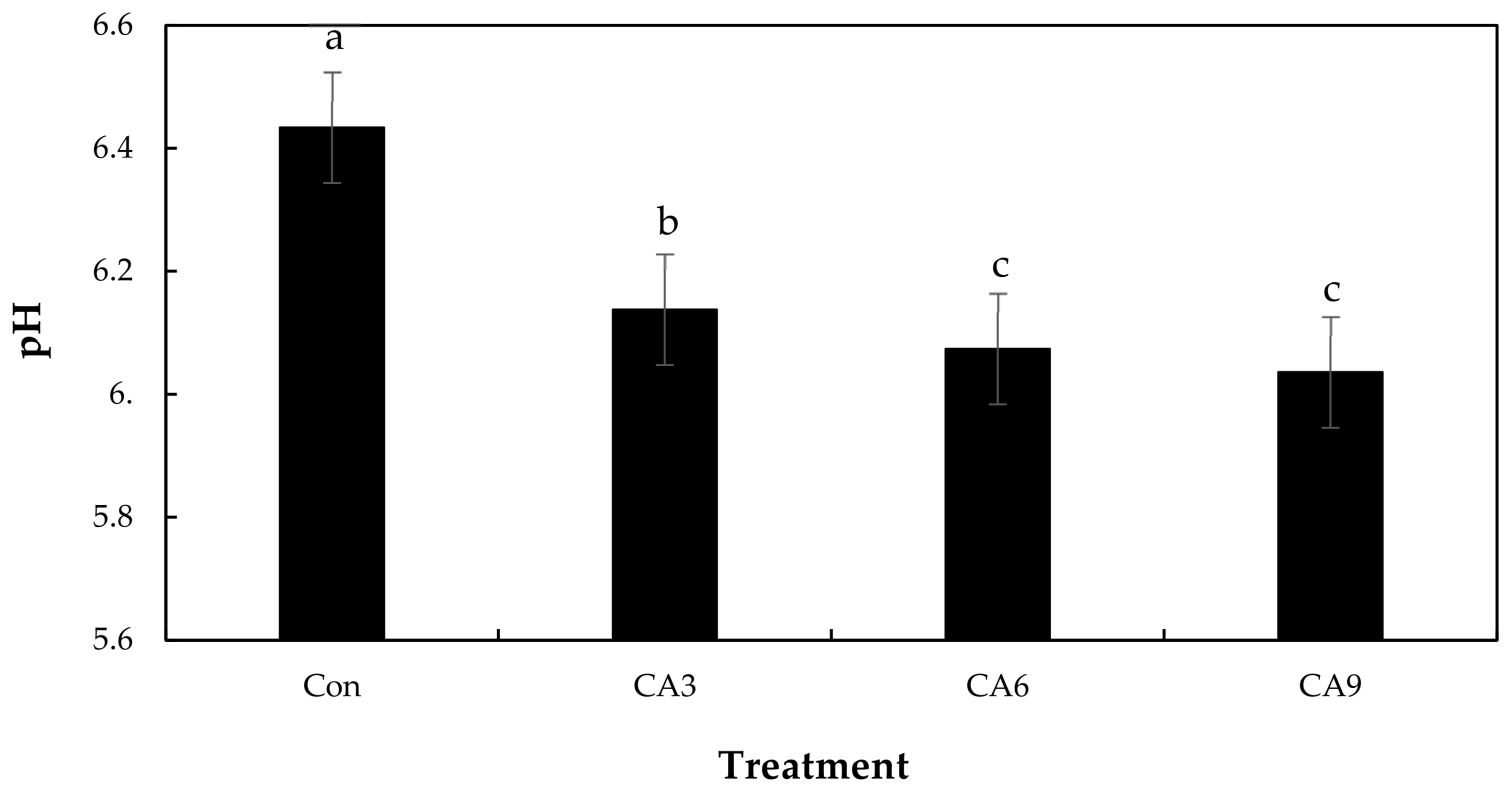
Active acidity (pH) of flavored butter supplemented with yuzu powder and almond granules. a–c Means in the bars with different letters are significantly different (*p* < 0.05). Con: unsupplemented butter; CA3: butter with 1% yuzu powder and 3% almond granules; CA6: butter with 1% yuzu powder and 6% almond granules; CA9: butter with 1% yuzu powder and 9% almond granules. Samples were prepared in three independent batches, and each batch was measured in triplicate.

The active acidity (pH) values of the flavored butter formulations are presented in Figure 2. All supplemented formulations had significantly lower pH values than Con (*p* < 0.05). CA6 had a lower pH than CA3, whereas CA6 and CA9 did not differ significantly. Among the supplemented formulations, pH tended to decrease as the almond level increased under the fixed yuzu concentration.

Previous studies reported decreased pH in foods supplemented with yuzu-derived ingredients [12,21]. Accordingly, the lower pH values of the supplemented formulations may be associated with the combined incorporation of yuzu powder and almond granules. However, the individual contributions of the two ingredients could not be determined because single-ingredient treatments were not included. Furthermore, the pH values of the ingredients were not measured directly in the present study.

### 3.3. Color

The color characteristics of the flavored butter formulations are summarized in Table 2.

Con had the highest lightness (L*) value (*p* < 0.05), followed by CA3, whereas CA6 and CA9 did not differ significantly. Redness (a*) did not differ significantly among the formulations. Yellowness (b*) increased in the order Con < CA3 < CA6 < CA9 (*p* < 0.05). Similar decreases in lightness have been reported in foods containing almonds or almond skins [22,23,24]. Differences between Con and the supplemented formulations may reflect the combined colors of yuzu powder and almond granules. The progressive reduction in lightness and increase in yellowness among CA3, CA6, and CA9 were associated with increasing almond levels under the fixed yuzu concentration. Because pigment concentrations were not measured, the contributions of specific pigments could not be confirmed. Chroma (C*ab) increased in the order Con < CA3 < CA6 < CA9 (*p* < 0.05), indicating a progressive increase in color saturation across the supplemented formulations. Hue angle (h°) did not differ significantly among the formulations (*p* = 0.8146), indicating that the overall hue direction remained similar despite changes in lightness and chroma. The total color difference (ΔE*ab), calculated relative to Con, was 7.19, 10.69, and 11.39 for CA3, CA6, and CA9, respectively. ANOVA performed only on these three supplemented formulations indicated a significant treatment effect (*p* = 0.0003). CA9 showed a greater color difference than CA3 and CA6 (*p* < 0.05), whereas CA3 and CA6 did not differ significantly. Thus, increasing almond addition under the fixed yuzu concentration intensified the overall color difference, primarily through reduced lightness and increased yellowness and chroma.

### 3.4. Viscosity

**Figure 3:**
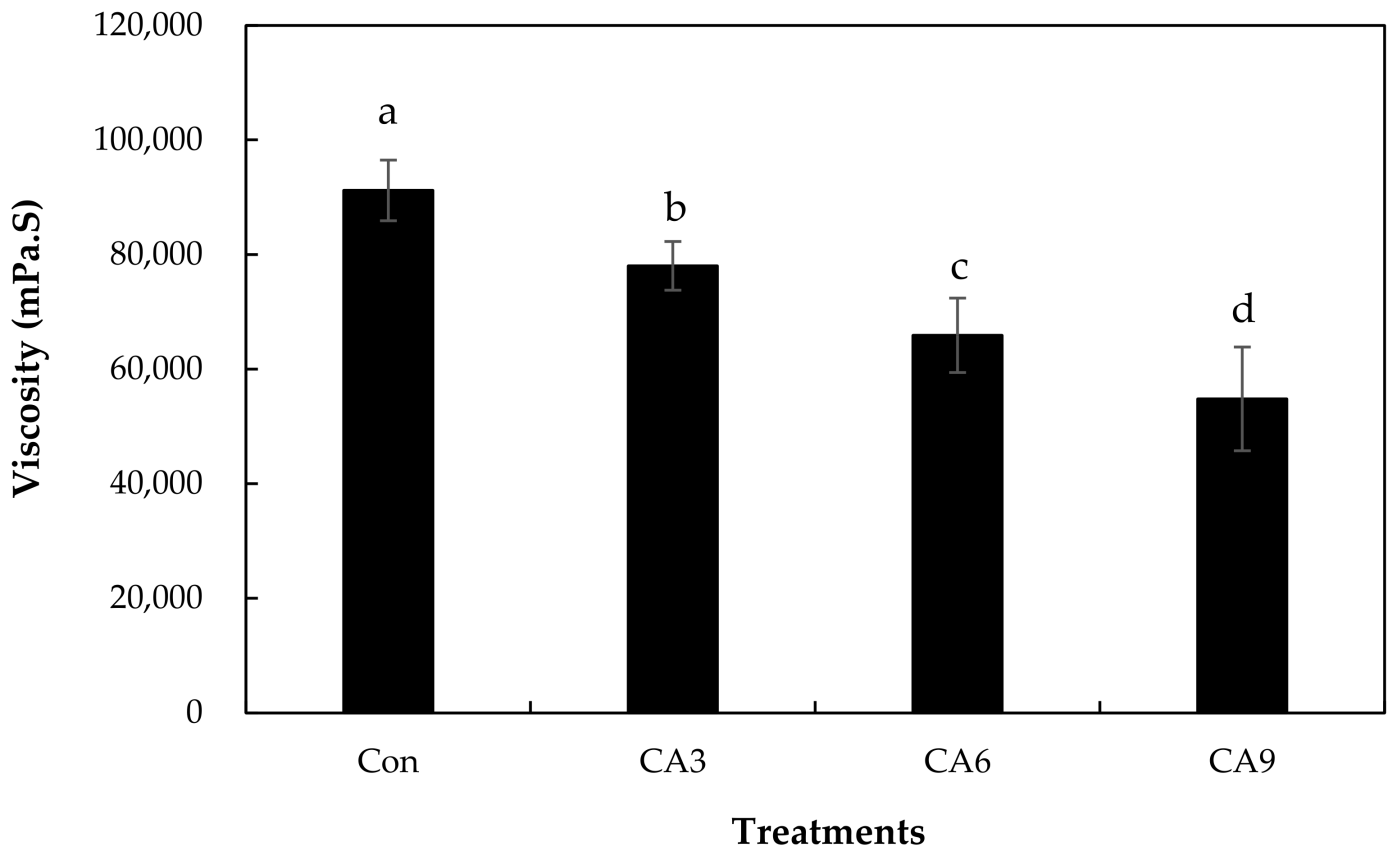
Viscosity of flavored butter supplemented with yuzu powder and almond granules. a–d Means in the bars with different letters are significantly different (*p* < 0.05). Con: unsupplemented butter; CA3: butter with 1% yuzu powder and 3% almond granules; CA6: butter with 1% yuzu powder and 6% almond granules; CA9: butter with 1% yuzu powder and 9% almond granules. Samples were prepared in three independent batches, and each batch was measured in triplicate.

The viscosity of the flavored butter formulations is presented in Figure 3. Con had the highest viscosity (*p* < 0.05), and viscosity decreased significantly across CA3, CA6, and CA9 as the almond level increased under the fixed yuzu concentration. The lower viscosity of the supplemented formulations may reflect combined differences in moisture, fat content, dispersed solid particles, and the overall butter matrix.

Milk-fat content and crystallization behavior can influence the spreadability and rheological properties of butter [25,26]. However, fat crystal structure and water-holding capacity were not measured in the present study. Therefore, weakening of the milk-fat network should be regarded as a possible explanation rather than a demonstrated mechanism. The observed decrease in viscosity was associated with increasing almond levels among CA3, CA6, and CA9, but differences between Con and the supplemented formulations reflect the combined effects of the two ingredients and processing.

### 3.5. Electronic Nose

**Figure 4:**
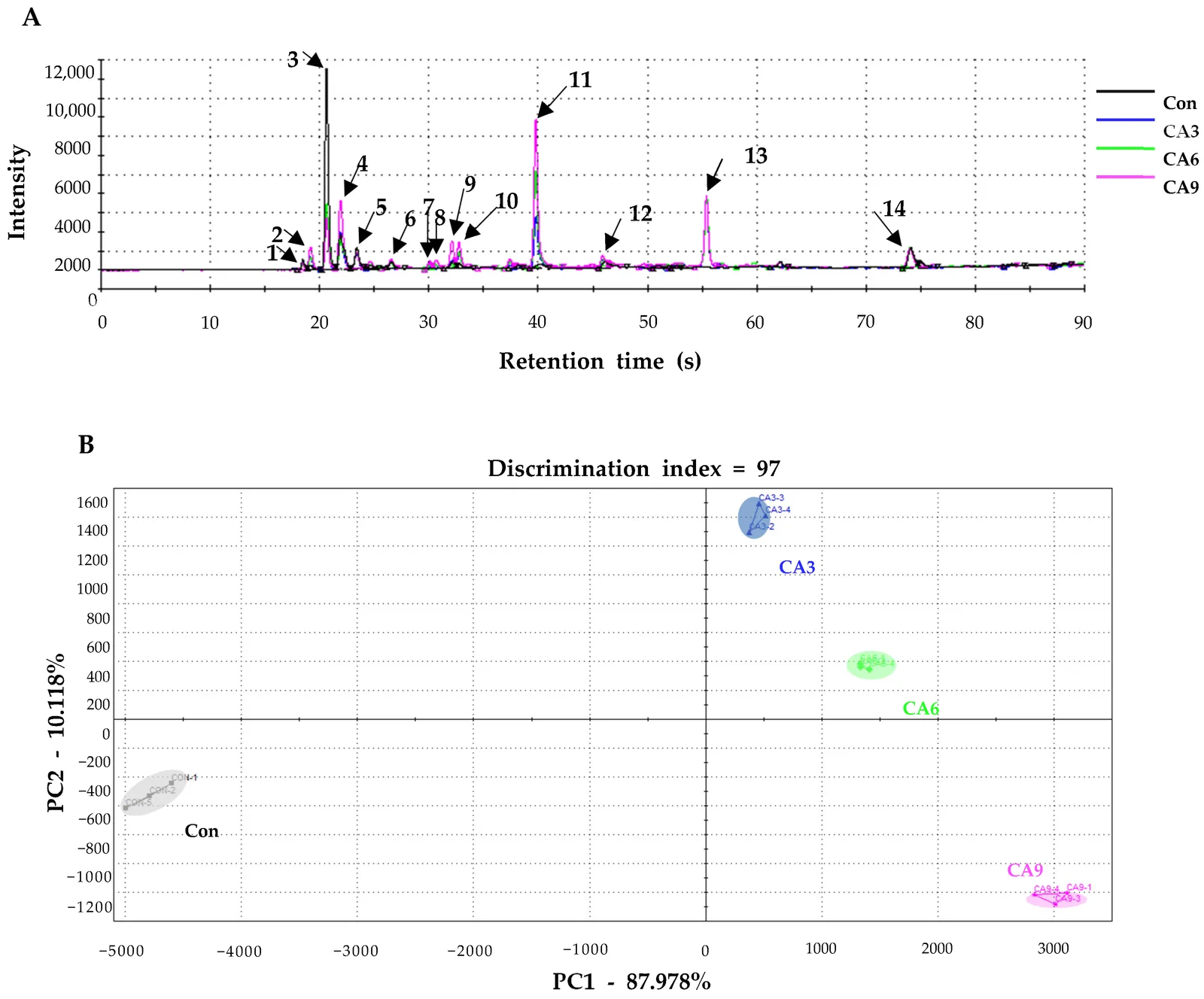
Peak chromatogram for aroma profiles (**A**) and principal component analysis (PCA) plot (**B**) of flavored butter supplemented with yuzu powder and almond granules. Con: unsupplemented butter; CA3: butter with 1% yuzu powder and 3% almond granules; CA6: butter with 1% yuzu powder and 6% almond granules; CA9: butter with 1% yuzu powder and 9% almond granules. Samples were prepared in three independent batches, and each batch was measured in triplicate.

The volatile profile patterns obtained by electronic-nose analysis are presented in Figure 4.

Electronic-nose analysis was used to compare volatile profile patterns among the butter formulations. Con showed relatively high intensities for peaks 3 and 11, which were tentatively assigned as propanal and butanoic acid, respectively, based on the instrument library. Peaks 2, 4, 7, 8, 9, 10, 11, and 13 were tentatively assigned as methanethiol, dimethyl sulfide, 3-methylbutanal, n-butanol, pentanal, 2,3-pentanedione, butanoic acid, and γ-terpinene, respectively. The relative intensities of these peaks varied among the formulations and generally increased across CA3, CA6, and CA9. This pattern was associated with increasing almond levels under the fixed yuzu concentration; however, the individual origins and sensory contributions of the peaks could not be confirmed without GC-MS, GC-IMS, or authentic standards [27,28].

The PCA score plot derived from the electronic-nose data is presented in Figure 4. The discrimination index was 97, indicating clear separation of the volatile profile patterns among samples. PC1 and PC2 explained 87.978% and 10.118% of the total variance, respectively. Along PC1, Con was separated from the supplemented formulations. Differences were also observed among CA3, CA6, and CA9, although these were smaller than the separation between Con and the supplemented formulations. Thus, the supplemented formulations had volatile profile patterns distinct from Con, and variation among CA3, CA6, and CA9 was associated with increasing almond levels under the fixed yuzu concentration. Electronic-nose analysis demonstrates pattern discrimination but does not confirm the identities or sensory effects of individual volatile compounds.

### 3.6. Electronic Tongue

**Figure 5:**
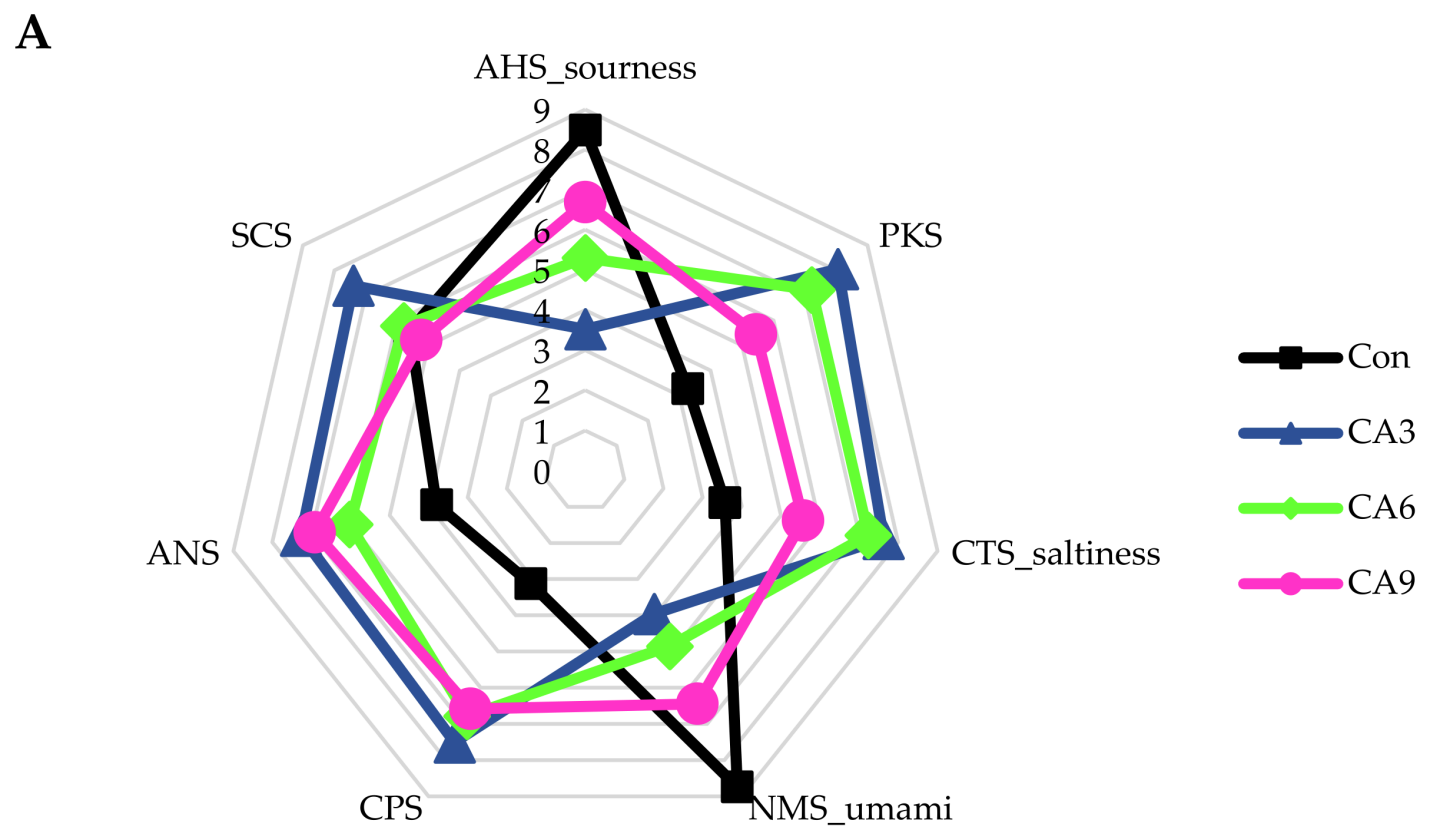

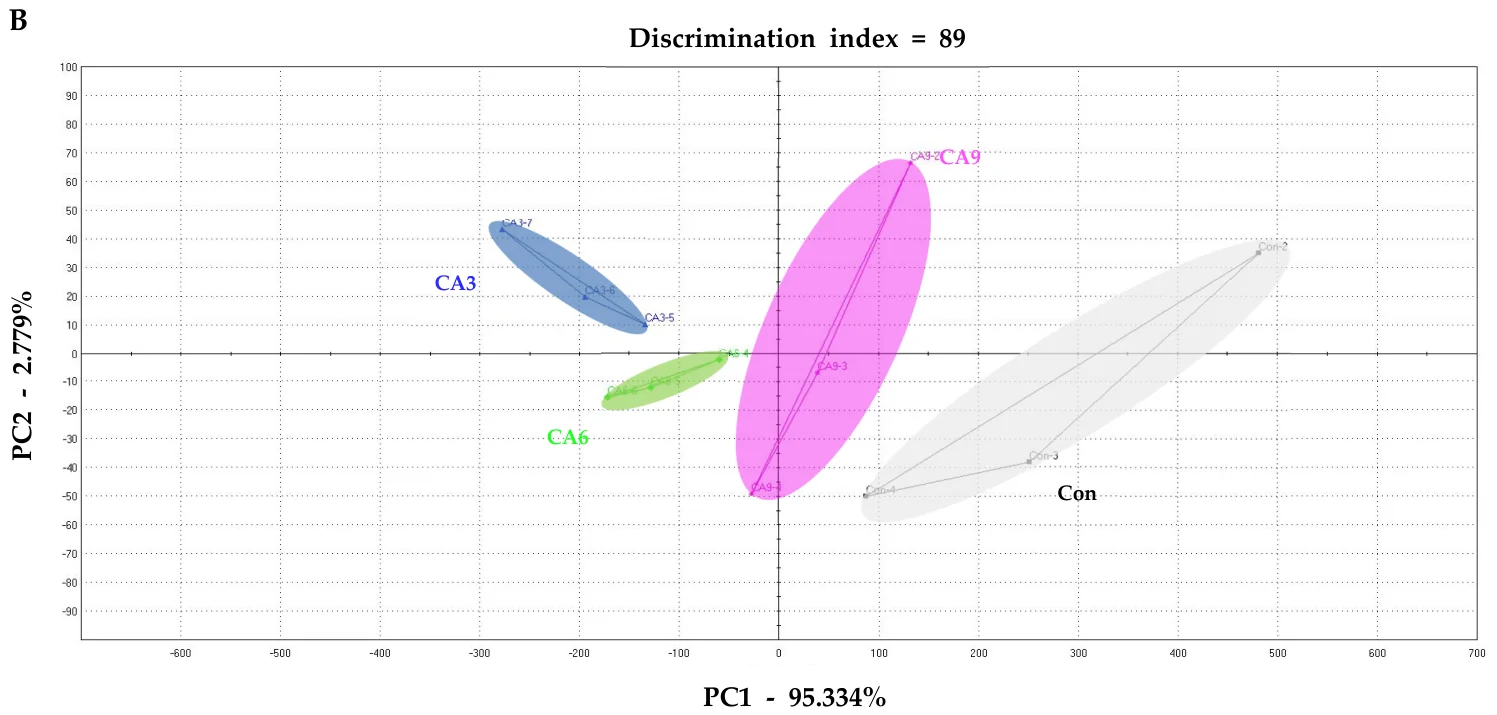
Radial graph for taste attributes (**A**) and principal component analysis (PCA) plot (**B**) of flavored butter supplemented with yuzu powder and almond granules. Con: unsupplemented butter; CA3: butter with 1% yuzu powder and 3% almond granules; CA6: butter with 1% yuzu powder and 6% almond granules; CA9: butter with 1% yuzu powder and 9% almond granules. Samples were prepared in three independent batches, and each batch was measured in triplicate.

The electronic-tongue responses and PCA results are presented in Figure 5.

The AHS response, for which a higher value represents stronger sourness, decreased in the order Con > CA9 > CA6 > CA3. Accordingly, all supplemented formulations showed lower instrumentally measured sourness responses than Con. Among CA3, CA6, and CA9, the AHS response increased with the almond level under the fixed yuzu concentration. Although the supplemented formulations had lower pH values, their AHS responses were lower than that of Con. This apparent discrepancy indicates that active acidity (pH) and the electronic-tongue sourness response should not be interpreted interchangeably. Matrix composition and sensor-sample interactions may have influenced the AHS response; however, this explanation could not be confirmed because titratable acidity and individual organic acids were not measured. Umami was highest in Con, followed by CA9, CA6, and CA3. Saltiness was lowest in Con and relatively high in CA3 and CA6. Because instrumental taste responses can be affected by the overall food matrix, the higher saltiness responses should not be interpreted as evidence of higher sodium content [29,30]. Measurements of sodium, titratable acidity, and individual organic acids are required to clarify these patterns.

The PCA score plot derived from the electronic-tongue data is presented in Figure 5. The discrimination index was 89, indicating marked differences in instrumental taste profiles among samples. PC1 and PC2 explained 95.334% and 2.779% of the total variance, respectively. Along PC1, Con was separated from the supplemented formulations, and CA3, CA6, and CA9 were also separated from one another. These findings indicate that the supplemented formulations had instrumental taste profiles distinct from Con. Separation among CA3, CA6, and CA9 was associated with increasing almond levels under the fixed yuzu concentration; however, the independent contributions of yuzu powder and almond granules could not be distinguished.

### 3.7. Sensory Evaluation

**Figure 6:**
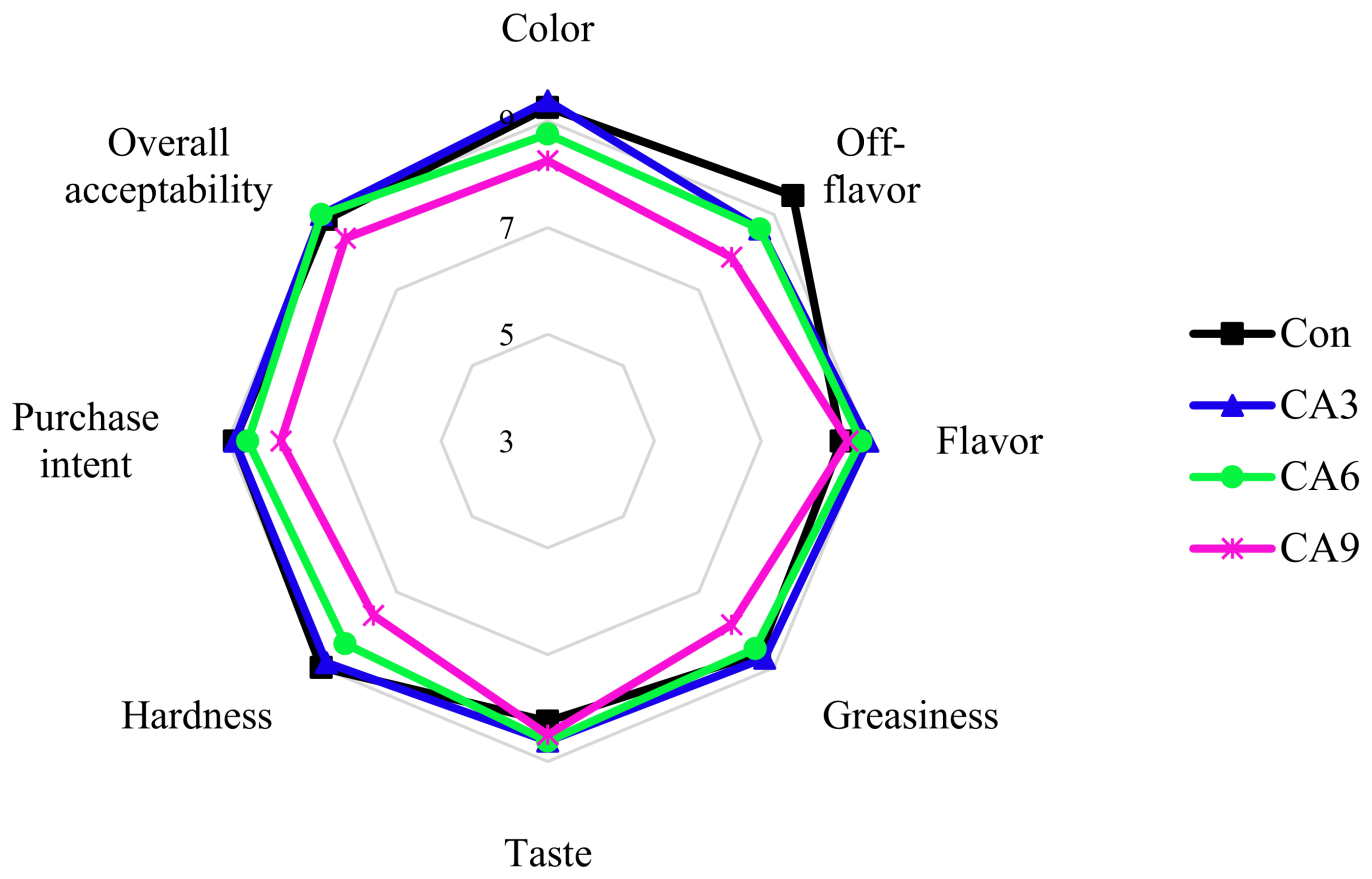
Spider plot of mean sensory scores for the flavored butter formulations. Con: unsupplemented butter; CA3, CA6, and CA9: butter with 1% yuzu powder and 3%, 6%, and 9% almond granules, respectively. Samples were prepared in three independent batches, and the sensory evaluation was conducted in three independent sessions.

The sensory evaluation results for the flavored butter formulations are presented in Table 3 and Figure 6.

For color, CA3 had a higher score than CA9 (*p* < 0.05), whereas Con and CA6 did not differ significantly from either sample. CA9 had the lowest numerical off-flavor score and had lower scores than Con and CA3 (*p* < 0.05), but did not differ significantly from CA6. CA3 had a higher flavor score than Con, CA6, and CA9 (*p* < 0.05), whereas the latter three formulations did not differ significantly. Greasiness scores did not differ significantly among the formulations (*p* = 0.2505). CA3 had the highest taste score and differed from all other formulations (*p* < 0.05). Con had a higher taste score than CA9 (*p* < 0.05), whereas CA6 did not differ significantly from Con or CA9. For hardness and purchase intent, CA9 had lower scores than Con and CA3, but did not differ significantly from CA6. Overall acceptability did not differ between CA3 and CA6, both of which had higher scores than Con and CA9. Con and CA9 did not differ significantly. Thus, CA3 and CA6 were comparable in overall acceptability, although CA3 showed favorable flavor and taste scores. The sensory profiles are visualized in Figure 6. The results suggest that the lower almond level in CA3 may have provided a favorable sensory balance under the fixed yuzu concentration [29,30,31]; however, this explanation remains hypothetical because the individual sensory contributions of the two ingredients were not separately evaluated.

This study has several limitations. First, because yuzu-only and almond-only treatments were not included, the independent and interactive effects of the two ingredients could not be determined. Therefore, differences between Con and the supplemented formulations should be interpreted as combined formulation effects. Second, the volatile peaks detected by the electronic nose were not confirmed using GC-MS, GC-IMS, authentic standards, or quantitative internal standards. Third, titratable acidity and individual organic acids were not measured, limiting interpretation of the discrepancy between active acidity (pH) and the electronic-tongue sourness response. Accordingly, the findings represent comparisons among the tested butter formulations rather than evidence of ingredient-specific, functional, or health-related effects.

## 4. Conclusions

This study compared the quality characteristics of an unsupplemented butter with flavored butter formulations containing 1% yuzu powder and 3–9% almond granules. The supplemented formulations had lower moisture and higher fat contents than Con, whereas ash content was highest in Con and did not increase proportionally with almond level. The supplemented formulations also differed from Con in active acidity (pH), color, and viscosity; among CA3, CA6, and CA9, viscosity decreased as the almond level increased. Electronic-nose analysis demonstrated differences in volatile profile patterns among the formulations, although individual compounds were not definitively identified. The electronic-tongue results showed that the sourness response decreased in the order Con > CA9 > CA6 > CA3. In the sensory evaluation, CA3 showed favorable flavor and taste scores, whereas CA3 and CA6 exhibited comparable overall acceptability. These findings suggest that lower-to-moderate almond levels may provide a favorable sensory balance in flavored butter containing 1% yuzu powder. However, because single-ingredient control groups were not included, the independent effects of yuzu powder and almond granules could not be determined. Further studies should employ a factorial experimental design and evaluate confirmed volatile compounds, fatty acid composition, lipid nutritional quality, oxidative stability, shelf life, and microbiological safety.

## Figures and Tables

**Table 1:** Proximate composition of flavored butter supplemented with yuzu powder and almond granules.

Proximate Composition (%)	Con	CA3	CA6	CA9	*p*-Value
Moisture	13.10 ± 1.16 ^a^	1.11 ± 0.35 ^c^	2.17 ± 0.45 ^bc^	2.89 ± 1.00 ^b^	<0.0001
Fat	85.25 ± 1.36 ^c^	98.64 ± 0.43 ^a^	97.53 ± 0.39 ^ab^	96.46 ± 1.26 ^b^	<0.0001
Ash	0.65 ± 0.08 ^a^	0.33 ± 0.07 ^b^	0.15 ± 0.10 ^c^	0.04 ± 0.03 ^c^	<0.0001

All values are expressed as the mean ± SD of three independent batches, with each batch analyzed in triplicate. a–c Means in the same row with different letters are significantly different (*p* < 0.05). Con: unsupplemented butter; CA3: butter with 1% yuzu powder and 3% almond granules; CA6: butter with 1% yuzu powder and 6% almond granules; CA9: butter with 1% yuzu powder and 9% almond granules.

**Table 2:** CIE color of flavored butter supplemented with yuzu powder and almond granules.

Traits	Con	CA3	CA6	CA9	*p*-Value
Lightness (L*)	61.14 ± 1.05 ^a^	55.04 ± 0.67 ^b^	51.59 ± 0.93 ^c^	51.44 ± 0.92 ^c^	<0.0001
Redness (a*)	3.67 ± 3.20	5.18 ± 3.09	5.63 ± 3.47	5.77 ± 3.60	0.6613
Yellowness (b*)	19.85 ± 0.41 ^d^	21.02 ± 0.71 ^c^	21.95 ± 0.26 ^b^	23.06 ± 0.84 ^a^	<0.0001
Chroma (C*ab)	20.27 ± 0.52 ^d^	21.83 ± 0.17 ^c^	22.85 ± 0.99 ^b^	23.99 ± 0.81 ^a^	<0.0001
Hue angle (h°)	80.04 ± 6.46	76.18 ± 8.32	75.89 ± 8.31	76.07 ± 8.62	0.8146
Color difference (ΔE*ab)	-	7.19 ± 1.45 ^b^	10.69 ± 1.24 ^b^	11.39 ± 0.83 ^a^	0.0003

All values are expressed as the mean ± SD of three independent batches, with each batch analyzed in triplicate. a–d Means in the same row with different letters are significantly different (*p* < 0.05). Con: unsupplemented butter; CA3: butter with 1% yuzu powder and 3% almond granules; CA6: butter with 1% yuzu powder and 6% almond granules; CA9: butter with 1% yuzu powder and 9% almond granules. Con was used as the reference for calculating ΔE*ab and was excluded from its statistical analysis. The ANOVA *p*-value and superscript letters for ΔE*ab refer only to comparisons among CA3, CA6, and CA9.

**Table 3:** Sensory evaluation of flavored butter supplemented with yuzu powder and almond granules.

Traits	Con	CA3	CA6	CA9	*p*-Value
Color	9.25 ± 0.82 ^ab^	9.37 ± 0.69 ^a^	8.75 ± 0.82 ^ab^	8.25 ± 0.96 ^b^	0.0385
Off-flavor	9.50 ± 0.70 ^a^	8.62 ± 1.21 ^a^	8.62 ± 1.11 ^ab^	7.87 ± 1.16 ^b^	0.0070
Flavor	8.50 ± 0.86 ^b^	9.00 ± 1.06 ^a^	8.87 ± 0.78 ^b^	8.62 ± 0.92 ^b^	0.0372
Greasiness	8.25 ± 0.96	8.62 ± 0.85	8.62 ± 0.69	8.50 ± 0.86	0.2505
Taste	8.62 ± 0.99 ^b^	8.75 ± 0.96 ^a^	8.50 ± 0.70 ^bc^	7.87 ± 0.78 ^c^	0.0006
Hardness	9.00 ± 1.00 ^a^	8.87 ± 0.92 ^a^	8.37 ± 0.85 ^ab^	7.62 ± 0.99 ^b^	0.0025
Purchase intent	8.87 ± 0.78 ^a^	8.87 ± 1.05 ^a^	8.62 ± 0.48 ^ab^	8.00 ± 0.70 ^b^	0.0405
Overall acceptability	8.87 ± 0.78 ^bc^	9.00 ± 0.86 ^a^	9.00 ± 0.50 ^a^	8.37 ± 0.69 ^c^	<0.0001

Values are presented as the mean ± SD of 24 individual scores per formulation, obtained from eight panelists over three evaluation sessions. a–c Means in the same row with different letters are significantly different (*p* < 0.05). Con: unsupplemented butter; CA3: butter with 1% yuzu powder and 3% almond granules; CA6: butter with 1% yuzu powder and 6% almond granules; CA9: butter with 1% yuzu powder and 9% almond granules.

## Data Availability

The original contributions presented in the study are included in the article, further inquiries can be directed to the corresponding author.
